# Etidronate-enhanced sodium hypochlorite irrigation in root canal therapy: A scoping review of efficacy and safety

**DOI:** 10.4317/jced.64364

**Published:** 2026-08-28

**Authors:** María Mihaela Iuga, Tatiana Ramírez-Mora, Stefan Vasile Stefanescu, Fernando Mauricio Espada-Salgado

**Affiliations:** 1 Faculty of Health Sciences, Private University of Tacna, Tacna, Peru; 2 Restorative Department, Faculty of Dentistry, University of Costa Rica, San José, Costa Rica; 3 Top Smiles, Liverpool L6 4DU, Merseyside, UK

**Keywords:** Chelation therapy, endodontics, etidronic acid, root canal irrigants, sodium hypochlorite.

## Abstract

**Background:**

Continuous chelation with sodium hypochlorite (NaOCl) and etidronic acid (HEDP) has been proposed for root canal irrigation. However, its efficacy and safety compared with sequential regimens remain unclear. To map evidence on the efficacy and safety of NaOCl + HEDP in root canal treatment.

**Materials and Methods:**

A scoping review was conducted according to Joanna Briggs Institute guidance and reported following the Preferred Reporting Items for Systematic Reviews and Meta-Analyses extension for Scoping Reviews. Web of Science, Scopus, PubMed/MEDLINE, Embase, and the Virtual Health Library were searched up to October 31, 2025. Of 576 records, 10 studies were included (6 in vitro, 3 ex vivo, and 1 clinical). Data were charted on study design, irrigation protocols, comparators, antimicrobial effects, cleaning outcomes, material compatibility, biomarkers, and postoperative pain.

**Results:**

Preclinical studies showed antimicrobial activity comparable to or greater than NaOCl alone or sequential NaOCl followed by ethylenediaminetetraacetic or citric acid. Continuous chelation was associated with improved apical smear layer removal and dentinal tubule patency, particularly with sonic or ultrasonic activation. Chemical and material compatibility was generally acceptable. The only randomized clinical trial reported higher pain during the first 48 hours, but no differences in analgesic intake or later pain.

**Conclusions:**

Preclinical evidence suggests that NaOCl + HEDP may improve apical cleaning while maintaining antimicrobial activity and acceptable compatibility. However, because the evidence is predominantly laboratory-based and only one clinical trial was identified, clinical superiority and safety cannot yet be established. Further randomized clinical trials are needed.

## Introduction

Sodium hypochlorite (NaOCl) remains the main irrigant in root canal treatment because of its tissue-dissolving capacity and broad antimicrobial activity [1,2]. However, it does not remove the inorganic component of the smear layer and is therefore commonly followed by a chelating agent such as ethylenediaminetetraacetic acid (EDTA) [1,3]. Although this sequential approach is effective, it adds clinical steps and may contribute to dentin erosion, reduced microhardness, and loss of available chlorine [3,4].

To simplify irrigation protocols, continuous chelation with etidronic acid (HEDP) mixed with NaOCl has been proposed as a one-solution strategy that allows deproteinization and chelation to occur simultaneously [3]. Previous studies have shown that this approach can maintain antimicrobial activity against infected dentin models [5,6] and remain compatible with ultrasonic activation and tissue-dissolving action under simulated endodontic conditions [7]. Additional evidence suggests that combining NaOCl with HEDP may enhance intracanal antibacterial effects when compared with some conventional irrigation approaches [8]. Studies have also reported improved smear layer and debris removal, particularly when activation methods are used [9].

A previous scoping review synthesized the laboratory evidence on continuous chelation available up to September 2022, focusing primarily on antimicrobial activity, smear layer and hard-tissue debris removal, and dentin erosion [10]. However, subsequent preclinical and clinical studies have evaluated additional outcomes, including interactions with endodontic materials, biological markers, and postoperative pain.

Despite these promising findings, the available evidence remains heterogeneous and fragmented. Important uncertainties persist regarding the magnitude of the antimicrobial effect compared with conventional sequential regimens, the influence of concentration and contact time, the interaction with dentin and endodontic materials, and the translation of laboratory findings into clinically relevant outcomes. Therefore, the aim of this scoping review was to map and synthesize the available evidence on the efficacy and safety of continuous chelation with NaOCl mixed with HEDP for root canal irrigation, compared with NaOCl alone, sequential NaOCl-EDTA protocols, or other relevant irrigation regimens across laboratory and clinical settings.

## Materials and Methods

*Design and reporting*

This scoping review was conducted in accordance with the Joanna Briggs Institute (JBI) methodological guidance for scoping reviews and is reported following the PRISMA extension for scoping reviews (PRISMA-ScR) checklist [11,12]. The protocol was registered a priori in the Open Science Framework (OSF; registration code: 8aef2; https://osf.io/8aef2). No deviations from the registered protocol were made. The completed PRISMA-ScR checklist is provided as Supplementary Material S1 (https://www.medicinaoral.com/medoralfree01/64364/Suppl1.pdf).

*Review question and PCC framework*

The review question was developed according to the Population-Concept-Context (PCC) framework.

Population: Extracted human teeth used in *in vitro* or *ex vivo* studies, and patients included in clinical studies; in situ human models were also considered when eligible.

Concept: Irrigation using sodium hypochlorite (NaOCl) mixed with etidronic acid, also reported as 1-hydroxyethylidene-1,1-bisphosphonate (HEBP), HEDP, or etidronate, and referred to as continuous chelation, one-solution, or single-solution irrigation.

Context: Root canal treatment in laboratory and clinical settings.

Eligible studies were those published between January 1, 2000 and October 31, 2025 that evaluated a mixed NaOCl + HEDP irrigation protocol. Studies assessing only sequential regimens without a mixed NaOCl + HEDP arm were excluded.

*Information sources and search strategy*

A comprehensive search was conducted in Web of Science, Scopus, PubMed/MEDLINE, Embase, and the Virtual Health Library (Biblioteca Virtual en Salud [BVS]), including Latin American and Caribbean Health Sciences Literature (LILACS), on October 31, 2025, without language restrictions. The search strategy combined controlled vocabulary and free-text terms related to etidronic acid, sodium hypochlorite, and endodontic irrigation. The complete database-specific search strategies, including field codes, controlled vocabulary, Boolean operators, date and document-type limits, and the number of records retrieved from each information source, are provided in Supplementary Material S2 (https://www.medicinaoral.com/medoralfree01/64364/Suppl2.pdf) and the OSF repository.

For transparency, the simplified Boolean search string was as follows: (etidronic acid OR etidronate OR HEDP OR HEBP OR "1-hydroxyethylidene-1,1-bisphosphonate") AND ("sodium hypochlorite" OR NaOCl) AND (endodont* OR "root canal" OR "root canal therapy" OR "root canal irrig*")

A total of 576 records were identified: Web of Science (n=124), Scopus (n=112), PubMed/MEDLINE (n=139), Embase (n=99), and BVS/LILACS (n=102). After removal of 391 duplicates, 185 records remained for screening. Sixteen full-text reports were assessed for eligibility, and 10 studies were included. Full-text exclusions were due to animal studies or non-human substrates (n=2), absence of a mixed NaOCl + HEDP arm (n=1), and studies outside the PCC framework, including solution- or instrument-only studies without a human dental substrate or clinical context (n=3).

*Study selection and inter-reviewer agreement*

After duplicate removal, two reviewers independently screened the titles and abstracts of 185 records using the predefined eligibility criteria. Potentially eligible reports were subsequently assessed independently in full text by the same reviewers. Disagreements were resolved through discussion and consensus; when consensus could not be reached, a third reviewer acted as adjudicator. Inter-reviewer agreement was quantified using percentage agreement and Cohen’s kappa coefficient before consensus or third-reviewer adjudication. Inclusion required the presence of a mixed NaOCl + HEDP study arm. Reasons for exclusion at the full-text stage were recorded in accordance with PRISMA-ScR recommendations.

*Data charting*

Data were extracted using a standardized charting form. The following information was collected: Year of publication, country, study design or experimental model, sample size, NaOCl and HEDP concentrations, application or exposure time, temperature, activation protocol, comparator groups, and reported outcomes. Funding sources, material or institutional support, and conflict-of-interest declarations reported by the included studies were also extracted. These data are presented in Supplementary Material S4 (https://www.medicinaoral.com/medoralfree01/64364/Suppl4.pdf).

The outcomes of interest were grouped into two domains. Efficacy outcomes included microbial reduction, tissue dissolution, smear layer and debris removal, apical cleanliness, penetration associated with activation methods, and instrumentation time. Safety-related outcomes included available chlorine, proteolytic activity, dentin-related effects such as microhardness, roughness, and erosion, interactions with endodontic materials and adhesion, biological responses, postoperative pain, analgesic consumption, and reported adverse events. Data charting was performed independently by two reviewers and cross-checked for accuracy. The extraction template and completed dataset are available in the OSF repository.

*Synthesis of results*

A descriptive narrative and tabular synthesis were performed because of the methodological heterogeneity across the included studies. Findings were organized according to outcome domain (efficacy and safety-related outcomes), study type (*in vitro*, *ex vivo*, in situ, or clinical), and comparator group. Whenever possible, results were further grouped according to root canal level (coronal, middle, or apical) and activation method (sonic, ultrasonic, or laser). No meta-analysis was performed.

*Critical appraisal*

In line with the purpose of a scoping review, no formal risk-of-bias assessment or certainty-of-evidence grading was undertaken [11,12]. Nevertheless, the methodological characteristics of the included studies were descriptively considered when interpreting the findings. These characteristics included sample size, appropriateness of comparator groups, reporting of irrigant concentration and contact time, standardization of activation protocols, blinding of outcome assessment when applicable, and the clinical relevance of the experimental model. This descriptive appraisal was not used as an eligibility criterion.

*Ethics and registration*

Ethical approval was not required because this study was based exclusively on published literature. The protocol was registered in the Open Science Framework. The supplementary materials comprise the completed PRISMA-ScR checklist (Supplementary Material S1: https://www.medicinaoral.com/medoralfree01/64364/Suppl1.pdf), complete database search strategies (Supplementary Material S2: https://www.medicinaoral.com/medoralfree01/64364/Suppl2.pdf), full-text eligibility decisions and inter-reviewer agreement analyses (Supplementary Material S3: https://www.medicinaoral.com/medoralfree01/64364/Suppl3.pdf), and funding and conflict-of-interest declarations for the included studies (Supplementary Material S4: https://www.medicinaoral.com/medoralfree01/64364/Suppl4.pdf).

## Results

*Study selection*

Database searches identified 576 records (Web of Science=124; Scopus=112; PubMed/MEDLINE=139; Embase=99; BVS/LILACS=102). After removing 391 duplicates, 185 records remained for title and abstract screening. At this stage, the reviewers initially agreed on 181 of the 185 records, corresponding to an observed agreement of 97.8%. Four disagreements were identified and subsequently resolved through discussion and third-reviewer adjudication. Cohen’s kappa was 0.863, indicating a high level of inter-reviewer agreement. After resolution of the disagreements, 16 reports were selected for full-text assessment.

During full-text assessment, the reviewers initially agreed on 15 of the 16 reports, corresponding to an observed agreement of 93.8%. One disagreement was resolved by the third reviewer. Cohen’s kappa was 0.862, also indicating a high level of inter-reviewer agreement. Following adjudication, 10 studies met the PCC eligibility criteria and were included, whereas six reports were excluded because they involved animal or non-human substrates (n=2), lacked a mixed NaOCl + HEDP arm (n=1), or were outside the PCC scope (n=3). The individual full-text decisions, reasons for exclusion, and detailed inter-reviewer agreement matrices are presented in Supplementary Material S3 (https://www.medicinaoral.com/medoralfree01/64364/Suppl3.pdf). These counts are shown in the PRISMA-ScR flow diagram (Figure 1).

*Characteristics of included studies*

The 10 included studies comprised *in vitro* (n=6), *ex vivo* (n=3), and clinical (n=1) designs involving human dentin or extracted teeth in laboratory models and patients in the clinical study. The only clinical study was a randomized clinical trial conducted in regenerative endodontic treatment (RET).

Continuous chelation involved NaOCl mixed with etidronate (HEDP), frequently using Dual Rinse HEDP, with NaOCl concentrations ranging from 1% to 5% and HEDP concentrations from approximately 9% to 18%. Comparators included NaOCl alone, sequential NaOCl followed by ethylenediaminetetraacetic acid (EDTA) or citric acid, and an alternative all-in-one irrigant. Activation protocols ranged from no activation to sonic or ultrasonic activation, including EDDY, passive ultrasonic irrigation (PUI), and XP-endo.

Outcomes included antimicrobial effects assessed by quantitative polymerase chain reaction (qPCR), confocal laser scanning microscopy (CLSM), and flow-biofilm models; smear layer and debris removal evaluated by scanning electron microscopy (SEM); material interactions and adhesion assessed by push-out bond strength and Fourier-transform infrared spectroscopy (FTIR); chemical outcomes related to available chlorine; biomarkers relevant to RET; and postoperative pain. Study design, sample characteristics, irrigant concentrations, application or exposure times, activation protocols, comparator groups, and outcomes are summarized in Table 1. Funding sources, material or institutional support, and conflict-of-interest declarations reported by the included studies are detailed in Supplementary Material S4 (https://www.medicinaoral.com/medoralfree01/64364/Suppl4.pdf).

*Synthesis by outcome domain*

Antimicrobial activity

In an *ex vivo* quantitative polymerase chain reaction (qPCR) study using infected premolars, continuous chelation with NaOCl + HEDP resulted in lower bacterial counts than NaOCl alone after chemomechanical preparation and supplementary agitation. After agitation, both HEDP- and citric acid-based regimens performed better than NaOCl alone [8].

In an *ex vivo* confocal laser scanning microscopy (CLSM) study using infected dentin segments, NaOCl + HEDP showed lower residual viable bacterial levels, including in cervical and deep dentin layers, than a sequential NaOCl followed by EDTA regimen [13].

In a dual-species flow-biofilm model evaluated using optical coherence tomography (OCT), 5% NaOCl was more effective than 2% NaOCl, regardless of the presence of HEDP. Although the addition of HEDP slowed the initial rate of biofilm disruption, the final antibiofilm effect was comparable to that achieved with NaOCl at the same concentration [16].

The available preclinical evidence suggests that NaOCl + HEDP maintains antimicrobial activity that is comparable to or, in some experimental models, greater than that of NaOCl alone or sequential irrigation regimens. However, its initial rate of action may be slower, particularly at lower NaOCl concentrations.

*Smear layer and debris removal and dentinal tubule patency*

In premolars without activation, a higher-concentration mixture of 2% NaOCl + 18% HEDP achieved better smear layer removal in the apical third than the lower-concentration mixture and control groups. However, complete apical cleanliness was not achieved in any group [15].

In premolars subjected to sonic activation, Dual Rinse HEDP + 3% NaOCl activated with EDDY produced the most favorable debris and apical smear layer scores. Even without activation, the mixed regimen performed better than the sequential NaOCl-EDTA-NaOCl protocol [9].

In infected dentin blocks, the presence of the smear layer reduced the antimicrobial performance of NaOCl alone, whereas NaOCl + HEDP maintained its activity and resulted in open dentinal tubules in approximately 95% of the evaluated specimens [6].

Preclinical findings suggest that continuous chelation may improve apical cleanliness and dentinal tubule patency. These effects appear to be enhanced when sonic or ultrasonic activation is used, although complete apical cleaning was not consistently achieved. A descriptive summary of the direction of effects across outcome domains is presented in Figure 2.

*Interactions with endodontic materials and adhesion*

Perforation repair cements. At 24 hours, NaOCl + HEDP resulted in the highest push-out bond strength for Biodentine and mineral trioxide aggregate (MTA). However, the differences among irrigation protocols were no longer observed after 21 days [14].

Root canal sealers. The all-in-one NaOCl + HEDP regimen resulted in higher push-out bond strength and a greater frequency of cohesive failures than Triton or saline. Fourier-transform infrared spectroscopy findings also indicated changes consistent with a potentially more favorable dentin-sealer interface [17].

The available laboratory evidence suggests that NaOCl + HEDP does not adversely affect the adhesion of the evaluated calcium silicate-based cements and root canal sealers. Early bond-strength advantages were observed in specific experimental models, but these findings cannot yet be generalized to clinical conditions.

*Chemical stability and available chlorine*

In an infected dentin model, the NaOCl + HEDP mixture maintained antimicrobial activity despite a modest reduction in available chlorine over 60 minutes. NaOCl alone showed greater chemical stability; however, the reduction in available chlorine in the mixed solution did not appear to eliminate its antimicrobial performance under the experimental conditions evaluated [13].

*Biological markers relevant to regenerative endodontic treatment*

In a regenerative endodontic treatment-like dentin-cylinder model, clodronate + NaOCl released more transforming growth factor beta 1 (TGF-β1) than etidronate + NaOCl or the sequential EDTA regimen. TGF-β1 release was comparable between etidronate and EDTA. No differences were observed between calcium hydroxide and triple antibiotic paste medicaments within the irrigation protocols [18].

Etidronate + NaOCl showed a biological response comparable to that of the sequential EDTA regimen. However, the clinical significance of TGF-β1 release from this laboratory model remains uncertain.

*Clinical outcomes*

The only randomized clinical trial, conducted in immature molars undergoing regenerative endodontic treatment, reported higher early postoperative pain between 6 and 48 hours in the NaOCl + HEDP group than in the sequential EDTA- or citric acid-based groups. However, no differences were found in analgesic consumption, and pain levels became comparable among groups between days 3 and 7 [19].

Clinical evidence remains limited to a single randomized trial. The higher early postoperative pain represents a potential safety-related signal, although the absence of differences in analgesic consumption and later pain outcomes prevents definitive conclusions regarding the clinical safety of NaOCl + HEDP.

## Discussion

This scoping review synthesized the available preclinical and clinical evidence on continuous chelation with sodium hypochlorite mixed with etidronic acid (NaOCl + HEDP). Overall, the findings suggest that continuous chelation maintains antimicrobial activity and may improve smear layer removal, apical cleanliness, and dentinal tubule patency under experimental conditions [5-9]. However, these findings should be interpreted cautiously because only 10 studies met the eligibility criteria, nine of which were preclinical, and considerable heterogeneity was observed in irrigant concentrations, contact times, activation methods, experimental models, and outcome measurements.

The present review updates and broadens the evidence previously mapped by La Rosa *et al*. [10]. Their scoping review focused exclusively on laboratory studies identified through PubMed and Scopus up to September 2022 and primarily evaluated antimicrobial activity, smear layer and hard-tissue debris removal, and dentin erosion. In contrast, the present review updated the search through October 2025 across five information sources and incorporated more recent *in vitro*, *ex vivo*, and clinical evidence. It also mapped additional outcomes related to available chlorine, interactions with calcium silicate cements and root canal sealers, bond strength, transforming growth factor beta 1 release, postoperative pain, and analgesic consumption. Most importantly, it included a randomized clinical trial that provided preliminary patient-reported evidence. Therefore, the contribution of the present review is to update and extend the previous evidence map toward material compatibility, regenerative endodontic outcomes, and initial clinical findings rather than to replace the earlier laboratory synthesis.

The difference in the number of included studies between the two reviews should be interpreted in relation to their eligibility criteria. The present review required the presence of a mixed NaOCl + HEDP arm and a human dental substrate or clinical context. Consequently, solution-only, instrument-only, animal, and non-human-substrate studies were excluded. Thus, the smaller number of included studies reflects a more specific Population-Concept-Context framework rather than a less comprehensive search.

From a chemical perspective, the available evidence indicates generally acceptable short-term compatibility under laboratory conditions. Dual Rinse HEDP increased the surface tension of NaOCl and reduced available chlorine over time, although antimicrobial activity was maintained in the infected-dentin model evaluated [13]. Nevertheless, this finding should not be interpreted as evidence that all NaOCl-HEDP formulations have equivalent chemical stability or performance.

Regarding interactions with repair materials, NaOCl + HEDP was associated with greater early push-out bond strength for Biodentine and mineral trioxide aggregate. However, the differences between irrigants were no longer observed after 21 days [14]. These findings suggest that continuous chelation did not adversely affect the adhesion of the calcium silicate-based materials evaluated, although the early bond-strength advantage cannot be directly extrapolated to clinical retention or long-term treatment success.

Continuous chelation was also associated with improved smear layer removal, particularly in the apical third, although complete apical cleanliness was not achieved in any experimental group [15]. These results indicate that HEDP may facilitate simultaneous organic and inorganic tissue removal, but they also confirm that irrigant chemistry alone is insufficient to completely clean anatomically complex apical areas.

Antimicrobial findings varied according to NaOCl concentration and the experimental model. In a dual-species biofilm model, 5% NaOCl was more effective than 2% NaOCl, regardless of HEDP addition. Although HEDP slowed the initial rate of biofilm disruption, the final antimicrobial effect was comparable to that obtained with NaOCl at the same concentration [16]. This suggests that NaOCl concentration and exposure time may influence antimicrobial performance more strongly than the distinction between continuous and sequential chelation.

Evidence concerning root canal sealers was also generally favorable. An *ex vivo* study reported greater push-out bond strength and a higher frequency of cohesive failures for epoxy resin-based and hydraulic calcium silicate-based sealers after the NaOCl + HEDP protocol than after an alternative all-in-one irrigant or saline [17]. However, these results were obtained under controlled experimental conditions and should be interpreted as preliminary evidence of material compatibility rather than proof of improved clinical sealing or treatment survival.

In a regenerative endodontic treatment-like model, etidronate + NaOCl produced transforming growth factor beta 1 release comparable to that obtained with the sequential EDTA regimen, whereas clodronate + NaOCl resulted in greater release [18]. Although growth-factor release may be biologically relevant to regenerative procedures, the clinical implications of this laboratory biomarker remain uncertain. Increased biomarker release cannot be assumed to result in improved tissue regeneration, root development, or clinical success.

Clinical evidence was limited to one randomized clinical trial involving regenerative endodontic treatment. The study reported higher postoperative pain during the first 6-48 hours with NaOCl + HEDP than with sequential EDTA- or citric acid-based protocols. Nevertheless, analgesic consumption did not differ between groups, and pain levels became comparable after 3-7 days [19]. The higher early pain should be considered a potential safety-related signal. However, a single trial with a limited sample cannot establish either clinical inferiority or a definitive safety profile.

Compatibility between NaOCl and HEDP may also vary according to the specific formulation and the presence of proprietary additives [20]. Therefore, future studies should report the exact composition of the NaOCl solution, HEDP concentration, preparation method, storage conditions, and interval between solution preparation and clinical use.

The effectiveness of apical irrigation depends not only on irrigant chemistry but also on canal preparation, apical enlargement, irrigation depth, solution exchange, and activation strategy [21]. These factors may partly explain the variability observed across the included studies and should be standardized when comparing continuous and sequential chelation protocols.

Recent *ex vivo* evidence has also suggested that continuous chelation may preserve fracture resistance, flexural strength, and microhardness of root dentin better than sequential NaOCl-EDTA irrigation [22]. Although these findings are relevant to the safety-related assessment of dentin, they remain based on laboratory mechanical outcomes and require confirmation under clinical conditions.

Similarly, evidence from irrigation studies indicates that smear layer and debris removal are strongly influenced by fluid dynamics and adjunctive activation methods [23]. Accordingly, the favorable cleaning outcomes observed with NaOCl + HEDP should not be attributed exclusively to the chelating agent, particularly when sonic or ultrasonic activation was simultaneously used.

Confocal evidence has further suggested that continuous chelation may improve the dentinal tubule penetration of bioceramic sealers compared with sequential chelation protocols [24]. Nevertheless, greater sealer penetration is a surrogate laboratory outcome, and its relationship with long-term apical healing or tooth survival remains uncertain.

A descriptive appraisal of the included studies identified several methodological limitations. Sample sizes were generally small, and substantial variability was observed in NaOCl and HEDP concentrations, exposure times, temperatures, specimen characteristics, activation protocols, comparator groups, and outcome assessment methods. Blinding of outcome assessment was not consistently reported, and several studies used highly controlled experimental models that may not reproduce the anatomical, microbial, and clinical complexity of root canal treatment. Moreover, only one clinical trial was available, and it evaluated postoperative pain rather than periapical healing, tooth survival, regenerative success, or long-term treatment outcomes.

Although safety was included as a domain of this review, most safety-related evidence was derived from laboratory assessments of chemical stability, dentin properties, biological responses, and compatibility with endodontic materials. These outcomes provide useful preliminary evidence but should not be considered equivalent to direct clinical evaluation of adverse events. Therefore, the findings support generally acceptable chemical and material compatibility under the experimental conditions evaluated, while evidence regarding clinical safety remains insufficient.

Future studies should standardize NaOCl and HEDP concentrations, solution preparation, irrigation volume, contact time, temperature, delivery method, and activation protocols. Adequately powered randomized clinical trials should compare continuous and sequential chelation using patient-centered and long-term outcomes, including postoperative pain, analgesic use, adverse events, periapical healing, regenerative outcomes, tooth survival, and overall treatment success.

Taken together, continuous chelation represents a promising irrigation strategy based primarily on laboratory evidence. Nevertheless, the current findings should be interpreted as an updated map of potential efficacy and compatibility rather than as confirmation of clinical superiority or established clinical safety.

## Conclusions

Preclinical evidence suggests that continuous chelation with sodium hypochlorite and etidronic acid may improve apical smear layer removal and dentinal tubule patency while maintaining antimicrobial activity and generally acceptable chemical and material compatibility under experimental conditions. However, the available evidence is limited to 10 heterogeneous studies, including only one randomized clinical trial. The higher early postoperative pain observed in that trial represents a potential safety-related signal, although analgesic consumption and later pain outcomes did not differ. Therefore, clinical superiority and a definitive clinical safety profile cannot currently be established. Further standardized and adequately powered randomized clinical trials evaluating adverse events, patient-reported outcomes, healing, and long-term treatment success are required. 

## Supplementary Material

Supplementary 1

Supplementary 2

Supplementary 3

Supplementary 4

## Figures and Tables

**Table 1:** Characteristics of included studies on continuous chelation with sodium hypochlorite (NaOCl) and etidronic acid (HEDP).

**No.**	**Study (Author, Year)**	**Model & specimen**	**Irrigation protocol & comparators**	**Outcomes measured**	**Key findings**
1	Erik *et al*., 2019	*In vitro*; mandibular premolars (n=78)	2 % NaOCl + 18 % HEDP (1:1); compared with 1 % NaOCl + 9 % HEDP, EDTA, HEDP alone and saline	Smear removal (SEM)	Best apical smear removal; none fully cleaned; apical residues greatest
2	Campello *et al*., 2022	*Ex vivo*; infected mandibular premolars (n≈66)	2.5 % NaOCl + 9 % HEDP vs 2.5 % NaOCl alone and NaOCl + citric acid; XP‑endo activation	Bacterial reduction (qPCR)	Better bacterial reduction vs NaOCl; mix and citric acid similar after agitation; highest qPCR‑negative rates
3	Aoun *et al*., 2023	*In vitro*; single‑rooted mandibular premolars (n=75)	DualRinse + 3 % NaOCl with or without sonic activation; compared with NaOCl - EDTA - NaOCl (± activation) and saline	Debris & smear removal (SEM)	Activation with HEDP mix gives best debris/apical smear removal; mix without activation better than EDTA sequence; mid/coronal similar
4	Rebolloso de Barrio *et al*., 2021	*In vitro*; root slices with perforation filled with Biodentine or MTA (n≈240)	Surface exposure to 5.25 % NaOCl + 9 % HEDP vs NaOCl, EDTA or saline; assessed at 1 day and 21 days	Push‑out bond strength; failure mode	Highest bond strength at 24 h with mix; effect dissipated by day 21; Biodentine > MTA at 24 h
5	Giardino *et al*., 2019	*Ex vivo*; infected dentin segments (closed‑end)	5 % NaOCl + HEDP (5 min) vs 5 % NaOCl followed by 17 % EDTA (2 min)	Viable bacteria (CLSM); surface tension; available chlorine	Lowest viable bacteria with HEDP mix; higher surface tension; available chlorine decreases with time
6	Borges *et al*., 2022	*In vitro*; dual‑species biofilm on dentin disks under flow	2 % and 5 % NaOCl with or without HEDP (DualRinse); laminar flow for 8 min	Biofilm removal & disruption (OCT)	5 % more effective than 2 %; HEDP slows removal but final efficacy similar; highlights efficiency vs efficacy
7	Akyüz *et al*., 2025	Clinical RCT; RET cases (n=42)	1.5 % NaOCl + 9 % HEDP vs NaOCl-EDTA or NaOCl-citric acid; all with passive ultrasonic activation	Post‑operative pain scores; analgesic use	Higher early postoperative pain (6-48 h) with HEDP mix; analgesic consumption similar; pain equalises by 3-7 days
8	Morago *et al*., 2016	*In vitro*; infected dentin blocks with or without smear layer	2.5 % NaOCl + 9 % HEDP vs 2.5 % NaOCl; smear removed or retained	Dead bacteria (%);open tubules (%)	Maintains bactericidal activity irrespective of smear; mix opens ~95 % of tubules vs none with NaOCl
9	Kamath *et al*., 2025	*Ex vivo*; mid‑root slices with AH Plus or CeraSeal sealer	Dual Rinse HEDP (NaOCl + HEDP) vs Triton vs saline; 5 min exposures; adhesives tested separately	Push‑out bond strength; FTIR ratios	Highest bond strength with HEDP mix; cohesive failures predominated; Triton intermediate; saline lowest; mix increases carbonate/phosphate ratio
10	Sridhar *et al*., 2025	*In vitro*; dentin cylinders with CH or TAP	Continuous chelation: 1.5 % NaOCl + 9 % etidronate or 7.6 % clodronate vs sequential NaOCl → EDTA; medicaments CH vs TAP	TGF‑β1 release (ELISA)	Clodronate + NaOCl releases more TGF‑β1 than etidronate or EDTA; etidronate similar to EDTA; no CH vs TAP difference

**Figure 1:**
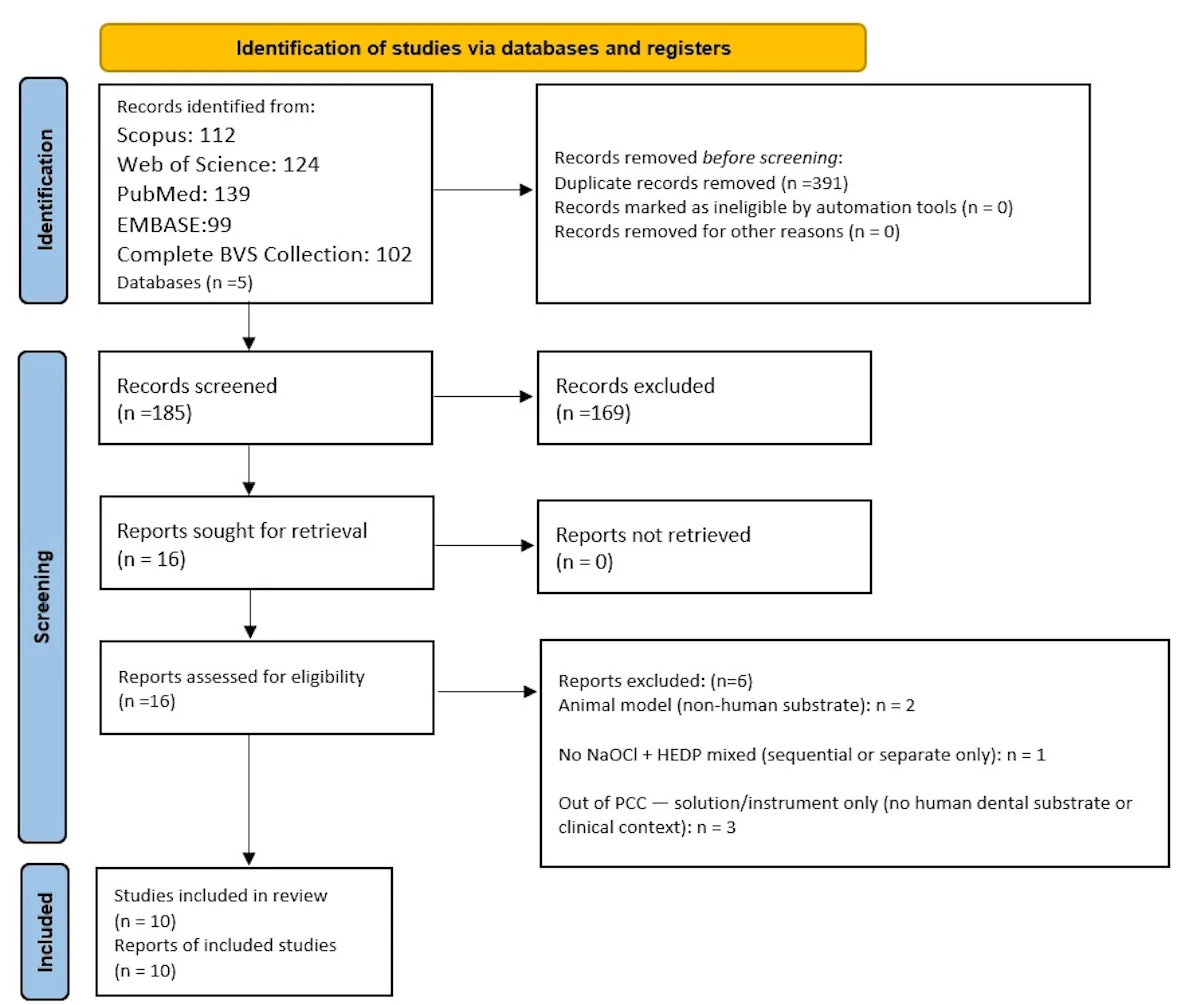
PRISMA-ScR flow diagram of study identification, screening, eligibility assessment, and inclusion.

**Figure 2:**
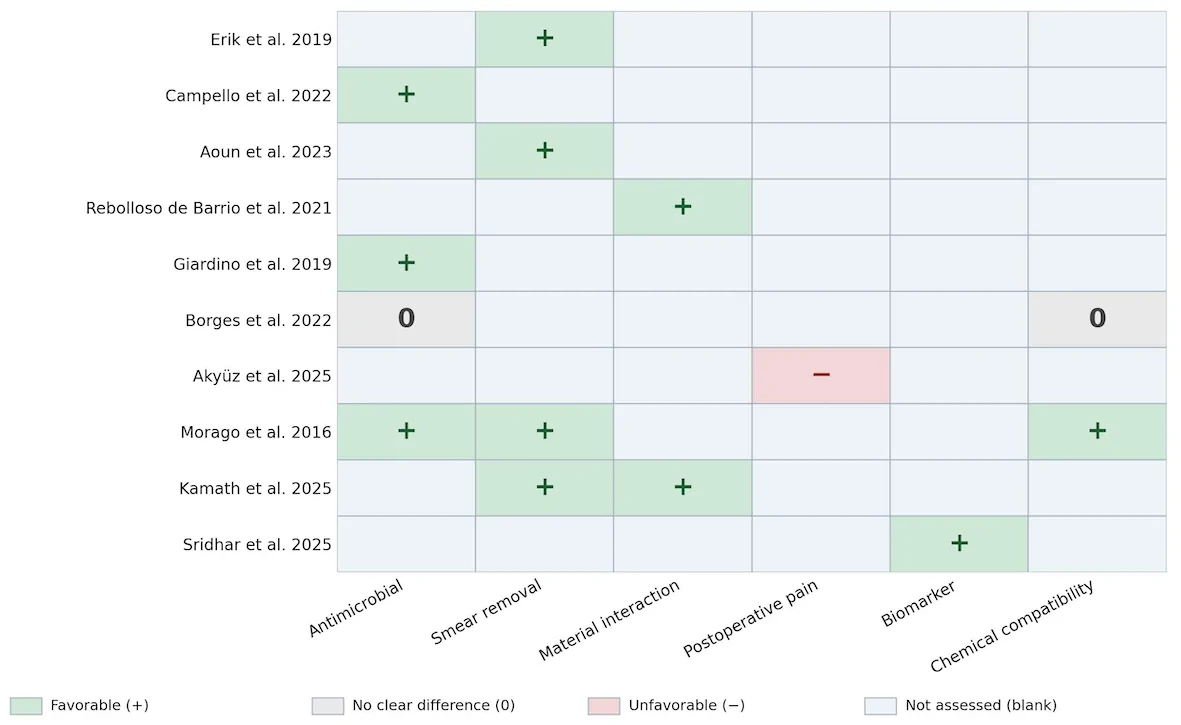
Qualitative evidence map showing the direction of effects of continuous chelation with NaOCl + HEDP. Symbols indicate the direction of the effect of NaOCl + HEDP relative to the main comparator in each study: (+) favorable, (0) no clear difference, (-) unfavorable, and blank when the outcome was not assessed. This figure provides a descriptive, non-quantitative summary of the available evidence.

## Data Availability

Declared none.
