# Supplementary material for: Etidronate-enhanced sodium hypochlorite irrigation in root canal therapy: A scoping review of efficacy and safety
Source: J Clin Exp Dent. 2026 Aug 28;18(9):e1113–22. doi: 10.4317/jced.64364 (PMC13677099; doi:10.4317/jced.64364)
Supplement: Supplementary file 2 [file jced-18-09-e1113-s002.pdf]

SUPPLEMENTARY MATERIAL S2

Complete Database Search Strategies

Etidronate-enhanced sodium hypochlorite irrigation in root canal therapy: a scoping review of efficacy and safety

Search date: October 31, 2025. **Coverage:** January 1, 2000 to October 31, 2025. **Language restrictions:** None. Controlled vocabulary and free-text terms were adapted to the syntax of each database. Date and document-type limits were applied through the database interface when they could not be expressed directly in the search string.

Table S2.1. Records identified by information source

Information source	Records identified
Web of Science Core Collection	124
Scopus	112
PubMed/MEDLINE	139
Embase	99
Virtual Health Library (BVS/LILACS)	102
**Total**	**576**

Scopus (Advanced Search)

```
TITLE-ABS-KEY(
  (
    (etidronic W/2 acid) OR etidronate OR HEDP OR HEBP OR
    "1-hydroxyethylidene-1,1-bisphosphonate"
  )
  AND ("sodium hypochlorite" OR NaOCl)
  AND (
    endodont* OR "root canal" OR "root canal therapy" OR
    "root canal treatment" OR "root canal irrig*" OR irrigat*
  )
)
AND PUBYEAR > 1999
AND PUBYEAR < 2026
AND DOCTYPE(ar OR re)
```

Limits: publication years 2000–2025; document type, article or review; no language restriction.

Web of Science Core Collection (Advanced Search)

```
TS=(
  (
    (etidronic NEAR/2 acid) OR etidronate OR HEDP OR HEBP OR
    "1-hydroxyethylidene-1,1-bisphosphonate"
  )
  AND ("sodium hypochlorite" OR NaOCl)
  AND (
    endodont* OR "root canal" OR "root canal therapy" OR
    "root canal treatment" OR "root canal irrig*" OR irrigat*
  )
)
```

Limits applied through the interface: publication years 2000–2025; document types, Article or Review; no language restriction.

PubMed/MEDLINE

```
(
  (
    "etidronic acid"[tiab] OR etidronate[tiab] OR HEDP[tiab] OR HEBP[tiab] OR
    "1-hydroxyethylidene-1,1-bisphosphonate"[tiab]
  )
  AND (
    "sodium hypochlorite"[MeSH Terms] OR "sodium hypochlorite"[tiab] OR NaOCl[tiab]
  )
  AND (
```

```
    endodont*[tiab] OR "root canal"[tiab] OR "root canal therapy"[tiab] OR
    "root canal treatment"[tiab] OR "root canal irrig*"[tiab] OR irrigat*[tiab] OR
    "Endodontics"[MeSH Terms] OR "Root Canal Irrigants"[MeSH Terms] OR
    "Root Canal Therapy"[MeSH Terms]
  )
)
AND ("2000/01/01"[Date - Publication] : "2025/10/31"[Date - Publication])
AND (Journal Article[Publication Type] OR Review[Publication Type])
```

No language restriction.

Embase (Elsevier; Emtree and free-text strategy)

```
(
  'etidronic acid'/exp OR 'etidronate'/exp OR
  (etidronic NEAR/2 acid):ti,ab,kw OR etidronate:ti,ab,kw OR
  HEDP:ti,ab,kw OR HEBP:ti,ab,kw OR
  "1-hydroxyethylidene-1,1-bisphosphonate":ti,ab,kw
)
AND (
  'sodium hypochlorite'/exp OR "sodium hypochlorite":ti,ab,kw OR NaOCl:ti,ab,kw
)
AND (
  'endodontics'/exp OR 'root canal irrigation'/exp OR
  endodont*:ti,ab,kw OR "root canal":ti,ab,kw OR
  "root canal therapy":ti,ab,kw OR "root canal treatment":ti,ab,kw OR
  "root canal irrig*":ti,ab,kw OR irrigat*:ti,ab,kw
)
AND [2000-2025]/py
AND (article OR review)/it
NOT 'conference abstract'/it
```

No language restriction; the final search was executed on October 31, 2025.

Virtual Health Library (BVS), including LILACS

```
tw:(
  (
    "etidronic acid" OR etidronate OR HEDP OR HEBP OR
    "1-hydroxyethylidene-1,1-bisphosphonate"
  )
  AND ("sodium hypochlorite" OR NaOCl)
  AND (
    endodont* OR "root canal" OR "root canal therapy" OR
    "root canal treatment" OR "root canal irrig*" OR
    endodoncia OR "canal radicular" OR
    "irrigación endodóntica" OR "irrigação endodôntica"
  )
)
```

Limits applied through the interface: publication years 2000–2025; type, article or review; no language restriction.

Search management

All retrieved records were exported to Zotero for deduplication and then imported into Rayyan for screening. The search yielded 576 records before duplicate removal.
