# Supplementary material for: Etidronate-enhanced sodium hypochlorite irrigation in root canal therapy: A scoping review of efficacy and safety
Source: J Clin Exp Dent. 2026 Aug 28;18(9):e1113–22. doi: 10.4317/jced.64364 (PMC13677099; doi:10.4317/jced.64364)
Supplement: Supplementary file 3 [file jced-18-09-e1113-s003.pdf]

SUPPLEMENTARY MATERIAL S3

Study Selection Details and Inter-reviewer Agreement

Etidronate-enhanced sodium hypochlorite irrigation in root canal therapy: a scoping review of efficacy and safety

This supplement presents the full-text eligibility decisions and the independent reviewer agreement calculated before consensus or third-reviewer adjudication.

Table S3.1. Full-text eligibility decisions

No.	Study	Brief topic	Decision	Reason
1	Akyüz et al. (2025)	Postoperative pain in regenerative endodontic treatment	Included	Clinical randomized trial; evaluated 1.5% NaOCl + 9% HEDP and a patient-reported outcome.
2	Morago et al. (2016)	Influence of smear layer on antimicrobial activity	Included	In vitro human-dentin model; evaluated a mixed NaOCl + HEDP protocol.
3	Erik and Özyürek (2018)	Cyclic fatigue of endodontic instruments	Excluded	Instrument-focused study without a human dental substrate or eligible endodontic treatment context.
4	Davril et al. (2025)	NaOCl–HEDP and mechanical properties of instruments	Excluded	Instrument-focused study without teeth or dentin; outside the PCC framework.
5	Kamath et al. (2025)	Dual Rinse HEDP and sealer adhesion	Included	Ex vivo human-root model; evaluated push-out bond strength after a mixed NaOCl + HEDP protocol.
6	Sridhar et al. (2025)	Continuous chelation and TGF-β1 release	Included	In vitro human-dentin model; evaluated NaOCl + etidronate and a biological marker.
7	Zollinger et al. (2018)	Stability of NaOCl mixed with HEDP	Excluded	Solution-only study without dental tissue or an eligible clinical/endodontic substrate.
8	Tartari et al. (2020)	Effect of heat on NaOCl with HEDP	Excluded	Animal-derived bovine tissue model; did not meet the human-substrate eligibility criterion.
9	Erik et al. (2019)	Smear layer removal with different etidronate concentrations	Included	In vitro extracted-human-tooth model; evaluated mixed NaOCl + HEDP protocols.
10	Campello et al. (2022)	Intracanal bacterial reduction with NaOCl + HEDP	Included	Ex vivo human-tooth model; compared the mixed protocol with relevant irrigation regimens.
11	Abdellatif et al. (2025)	Bovine pulp dissolution with Dual Rinse HEDP	Excluded	Bovine tissue model; did not meet the human-substrate eligibility criterion.
12	Aoun et al. (2023)	Dual Rinse HEDP with or without sonic activation	Included	In vitro extracted-human-tooth model; evaluated debris and smear layer removal.
13	Rebolloso de Barrio et al. (2021)	NaOCl + HEDP and adhesion of repair cements	Included	In vitro human-root-slice model; evaluated push-out bond strength.
14	Giardino et al. (2019)	Surface tension and dentin disinfection	Included	Ex vivo infected-human-dentin model; evaluated disinfection and available chlorine.
15	Khalil et al. (2025)	Dentin-related regenerative outcomes	Excluded	No mixed NaOCl + HEDP arm; the agents were evaluated separately.
16	Borges et al. (2022)	Dual-species biofilm response to NaOCl + HEDP	Included	In vitro human-dentin-disk model; evaluated real-time biofilm disruption.

Eligibility rule: studies were included when they evaluated a mixed NaOCl + HEDP/etidronate protocol in extracted human teeth, human dentin, or patients and reported an efficacy- or safety-related outcome.

Table S3.2. Inter-reviewer agreement during study selection

Screening stage	Records evaluated	Initial agreements	Disagreements	Observed agreement	Cohen's κ	Final included
Title and abstract screening	185	181	4	97.8%	0.863	16
Full-text assessment	16	15	1	93.8%	0.862	10

Note. Cohen's κ was calculated from the reviewers' independent decisions before consensus or third-reviewer adjudication.

Detailed 2 × 2 decision matrices

A. Title and abstract screening (n = 185)

Reviewer 1 / Reviewer 2	Include	Exclude	Total
**Include**	14	3	17
**Exclude**	1	167	168
**Total**	15	170	185

Observed agreement: 181/185 = 97.8%; Cohen's κ = 0.863.

B. Full-text assessment (n = 16)

Reviewer 1 / Reviewer 2	Include	Exclude	Total
**Include**	10	1	11
**Exclude**	0	5	5
**Total**	10	6	16

Observed agreement: 15/16 = 93.8%; Cohen's κ = 0.862.

Resolution of disagreements. Four title/abstract disagreements were resolved through discussion and, when required, third-reviewer adjudication. One full-text disagreement was resolved by the third reviewer.
