# Supplementary material for: Etidronate-enhanced sodium hypochlorite irrigation in root canal therapy: A scoping review of efficacy and safety
Source: J Clin Exp Dent. 2026 Aug 28;18(9):e1113–22. doi: 10.4317/jced.64364 (PMC13677099; doi:10.4317/jced.64364)
Supplement: Supplementary file 4 [file jced-18-09-e1113-s004.pdf]

SUPPLEMENTARY MATERIAL S4

Funding, Material Support, and Conflict-of-Interest Declarations of Included Studies

Etidronate-enhanced sodium hypochlorite irrigation in root canal therapy: a scoping review of efficacy and safety

Supplementary Table S4.1. Funding, material or institutional support, and conflict-of-interest declarations reported in the 10 included studies.

No.	Study	Funding or material/institutional support	Conflict-of-interest declaration
1	Erik et al., 2019	Funding was not reported in the article.	The authors declared no conflict of interest.
2	Campello et al., 2022	Supported by grants from Fundação Carlos Chagas Filho de Amparo à Pesquisa do Estado do Rio de Janeiro (FAPERJ) and Conselho Nacional de Desenvolvimento Científico e Tecnológico (CNPq), Brazilian governmental institutions.	The authors denied any conflicts of interest related to the study.
3	Aoun et al., 2023	The study reported that it did not receive financial support.	The authors denied any conflict of interest.
4	Rebolloso de Barrio et al., 2021	The research did not receive any specific grant from funding agencies in the public, commercial, or not-for-profit sectors.	The authors declared no competing interests.
5	Giardino et al., 2019	Funding was not reported in the article.	The authors declared no conflict of interest.
6	Borges et al., 2022	Mariana Maciel Batista Borges and Marco Antonio Hungaro Duarte were financially supported by a CAPES scholarship (PRINT/CAPES grant 88887.371170/2019-00).	The authors denied any conflicts of interest related to the study.
7	Akyüz et al., 2025	The study was self-funded. Open-access funding was provided by the Scientific and Technological Research Council of Türkiye (TÜBİTAK).	The authors declared no competing interests.
8	Morago et al., 2016	Supported by Research Group CTS-167, Junta de Andalucía, Spain.	The authors denied any conflicts of interest related to the study.
9	Kamath et al., 2025	Medcem GmbH supplied the Dual Rinse HEDP capsules, and Ajman University provided institutional support. No separate financial funding statement was reported.	No conflict of interest was declared.
10	Sridhar et al., 2025	Financial support and sponsorship: Nil.	The authors declared no conflicts of interest.

Note. Statements were transcribed or conservatively paraphrased from the funding, acknowledgments, financial disclosure, and conflict-of-interest sections of the included publications. "Funding was not reported" indicates that no explicit funding statement was identified in the article.
